# Chemotype of damask rose with oleic acid (9 octadecenoic acid) and its antimicrobial effectiveness

**DOI:** 10.1038/s41598-021-87604-1

**Published:** 2021-04-13

**Authors:** Mansureh Ghavam, Afsaneh Afzali, Maria Letizia Manca

**Affiliations:** 1 Department of Range and Watershed Management, Faculty of Natural Resources and Earth Sciences, University of Kashan, Kashan, Iran; 2 Department of Environment, Faculty of Natural Resources and Earth Sciences, University of Kashan, Kashan, Iran; 3 Department Life and Environmental Sciences, University of Cagliari, Cagliari, Italy

**Keywords:** Biochemistry, Biogeochemistry

## Abstract

Essential oils are natural products that have great antimicrobial potential value against many fungi and bacteria. *Rosa damascena* Mill. is one of the most important aromatic species of the Rosaceae family from which essential oil and economically valuable products can be obtained. The present study was designed to investigate the major compositions of the essential oil of this plant in Isfahan region of Iran and to identify its antibacterial and antifungal effects against 11 microorganisms causing human diseases and food spoilage. The essential oil was extracted by using the Clevenger apparatus and was analyzed by gas chromatography-mass spectrometry (GC–MS) technique. Its antimicrobial activity was evaluated by well diffusion, minimum inhibitory concentration (MIC) and minimum bactericide concentration (MBC). The results showed that the most important compounds of the essential oil were nonadecane (24.72%), heneicosane (19.325%), oleic acid (17.63%), and citronellol (12.61%). The results also showed that the highest inhibition zone of rose essential oil was against *Aspergillus brasiliensis* (15.00 ± 0.00 mm) and had a significant effect on *Klebsiella pneumoniae* (~ 8.00 mm). Also the rose oil had a significant inhibition and lethal effect against *Candida albicans* (MIC and MBC ~ 125 μg/mL), which is equivalent to the nystatin antibiotic (~ 125 μg/mL). Therefore, the essential oil of Damask rose can be considered as an alternative natural product for the prevention and treatment of fungal diseases in humans and against food spoilage as well.

## Introduction

Natural products, such as essential oils, have historically proven their value as a source of molecules with antimicrobial potential, and may have high potential in bacterial and fungal infections in the near future. Indeed, the synergistic interaction of essential oils with antimicrobials has been previously reported as a valid approach to prevent the emergence of multidrug resistance by restoring their antimicrobial effect and reducing adverse effects of synthetic antimicrobials^1^.

*Rosa damascena* Mill. (Damask rose), is one of the most important aromatic species of Rosaceae family from which essential oils and high economic value products can be obtained and widely used in various pharmaceuticals, food, perfume, and cosmetic industries^2^. Petals and jars of flowers are as a rich source of Vitamin C, and their essential oil have sedative properties and antiviral and antibacterial effects^3,4^. The most important components of the essential oil are citronellol, nonadecane, heneicosane, *β*-caryophyllene, geraniol, nerol, linalool, and phenyl ethyl acetate^5^.

The birthplace and starting habitat of Damask rose is the ancient land of Iran and the Middle East. Iran is one of the oldest rose oil producing country in the world and its progress in this field reaches more than 2500 years^6^. Essential oil composition of *R. damascena* is very diverse in different climates areas of Iran. Citronellol, nonadecane, and, geraniol are essential components of rose essential oils from the central part Iran^7^. While the chemical composition of this essential oil changed significantly in the northern of Iran as the main components are geraniol, 1-nonadecene, n-tricosane, and hexatriacontane^8^.

The chemical composition of the oil is influenced by the plant's origin, climatic conditions, and technology used to produce the oil^9^. The antimicrobial activity of essential oils in general, and of rose essential oil in particular, depends largely on their chemical composition^10^. Antibacterial activity of Rose Damascus essential oil against some bacteria such as *Propionibacterium acnes*^11^, *Escherichia coli*, *Proteus vulgaris*, *Klebsiella pneumoniae*, *Candida albicans*, *Enterococcus faecalis*, and S*taphylococcus aureus*^12^, *Bacillus subtilis* and *Streptococcus pyogenes*^13^, and *Bacillus cereus*, *Staphylococcus epidermidis*, and *Pseudomonas fluorescens*^10^ have been reported in different countries, but so far, the antibacterial and antifungal activity of this oil has not been investigated simultaneously on 11 strains of microorganisms. This study was performed for the first time to investigate the composition of essential oils obtained from Rose Damascus harvested from Isfahan region of Iran and its antibacterial and antifungal activity.

## Materials and methods

### Plant material

To maximize the yield of rose oil, flowers (petals and sepals) were harvested at 6 am from Isfahan province in May 2019 (Permission for collection of plant materials obtained from the Agricultural Jahad Office and also the owner of the farm). Three points were selected randomly and at each point, flowers were collected randomly from different plants (100 plants at each point). Samples were transferred to the laboratory after harvesting. A voucher specimen has been deposited in the herbarium of the Faculty of Natural Resources and Earth Sciences, University of Kashan, Kashan, Iran. The plant was identified by Mansureh Ghavam and recorded with code number 1310.

### Extraction of essential oil

All the plant experiments were carried out in accordance with guidelines. To isolate the essential oil, three hundred gram of fresh flowers for each replicate of each region were weighed and then extracted by water distillation using a Clevenger apparatus for 4 h. The essential oil was dehydrated by sodium sulfate and stored in dark bottles at 4 °C until further use. This process was repeated three times.

### GC–MS analyses of essential oil

The determination of the constituents of essential oil samples was done by Gas chromatography-mass spectrometry (GC–MS). Model 6890 chromatography coupled with Agilent model N-5973 mass spectrometer with HP-5MS capillary column with 5% Methylphenylsiloxane static phase (length 30 m, internal diameter 0.25 mm, layer static thickness 0.25 μm) and ionization energy of 70 eV was used for qualitative identification of components. In temperature programming for the analysis, the temperature would first start at 60 °C in the oven and then would rise at a rate of 3–246 °C. The temperature of injector and detector temperature were was 250 °C, the sample volume was 1 µl with 1.50 split and the helium carrier gas at a flow rate of 1.5 mL/min. The chemical components of the essential oils were identified based on the analysis of the chromatograms of each oil regarding the retention indices (RI) in relation to standards of n-alkane mixtures (C8-C20) and mass spectral data of each peak using a computer library (Wiley-14 and NIST-14 Mass Spectral Library), along with comparing these data with literature^14^.

### Antimicrobial activity assays of essential oil

Eleven microorganisms were used to evaluate the antimicrobial activity of the extract. The used microbial strains were provided by the Iranian Research Organization for Science and Technology (IROST). Four Gram-positive bacteria were *Staphylococcus epidermidis* (CIP 81.55), *Staphylococcus aureus* (ATCC 29737), *Streptococcus pyogenes* (ATCC 19615), and *Bacillus subtilis* (ATCC 6633) and the group of Gram-negative bacteria included *Klebsiella pneumonia*e (ATCC 10031), *Shigella dysenteriae* (PTCC 1188), *Pseudomonas aeruginosa* (ATCC 27853), *Salmonella paratyphi-A serotype* (ATCC 5702), and *Escherichia coli* (ATCC 10536). The used fungal strains were *Aspergillus brasiliensis* (ATCC 16404) and *Candida albicans* (ATCC 10231). The bacterial strains were cultured on nutrient agar and the fungal strains on Sabouraud dextrose agar (SDA). The plates inoculated with the bacteria and fungi cultures were incubated overnight at 37 °C and 30 °C, respectively.

This procedure was performed according to CLSI standards. For this purpose, plates containing Müller Hinton agar and SDA were prepared to culture the bacterial and fungal strains, respectively. The essential oil was dissolved in dimethylsulfoxide (DMSO) and a concentration of 30 mg/mL was reached. Then 100 µL of bacterial suspensions with a half-McFarland turbidity equivalent in culture medium were cultured in 6 mm well plates and 10 μL (at concentration 30 mg/mL) of essential oil was poured into the wells. The plates were incubated for 48 h and 72 h at 30 °C for fungal strains and for 24 h at 37 °C for bacterial strains. The antimicrobial activity was evaluated for each microorganism by measuring the inhibition diameter with the antibiogram ruler (in millimeters). To evaluate the repeatability of the results, three replicates were performed for each strain. Results were calculated as mean ± SD. Dimethylsulfoxide was used as negative control and gentamicin and rifampin antibiotics or the antifungal nystatin were used for bacteria and fungi respectively, to compare their inhibitory potency with that of essential oil.

The minimum concentration capable of inhibiting the growth was calculated for microbial susceptible to the extracts by means of microdilution method. In this method, various dilutions of the essential oil were prepared. A specific amount of sample was weighed and a suitable ratio of TSB medium and DMSO solvent were used to prepare the initial stock with the concentration of 2000 μg/mL. Then, serial dilutions to obtain lower concentrations (1000, 500, 250, 125, 62.5, 31.25, and 15.63 µg/mL) were prepared. For this purpose, sterile 96-well microplates were provided. To each plate 95 µL of culture medium, 5 µL of bacterial suspension with 0.5 McFarland dilution, and 100 µL of different essential oil dilutions were added, and then the plate was incubated at 37 °C for 24 h for bacterial strains and at 30 °C for 48 h for yeast. Based on the opacity and color change in each well, MIC or minimum concentration capable of inhibiting the growth was determined. To determine the minimum bacterial concentration (MBC), after 24 h incubation, wells containing non-growth medium were inoculated with nutrient agar medium and incubated at 37 °C for further 24 h. These tests were performed in triplicate.

### Statistical analysis

The statistical analysis was performed using SPSS software. First, the normality of the statistical variables was investigated using a Kolmogorov–Smirnov test, and after ensuring the normality of the data, the variance of the data was analysed using One-Way Analysis of Variance (ANOVA) with a probability level was 5% error was performed.

## Results and discussion

### Essential oil composition

The most important and abundant components of the isolated essential oil obtained from *R. damascena* are reported in Table 1. The results showed that there were 20 different components in the essential oil with a relative composition of 99.72%. Some difference have been detected among the different areas of Iran and in the world as 34 compounds (97%) were found in the specie harvested from the central part of Iran, 17 compounds (97.42%) from that of the northern of Iran, 8 compounds (99.98%) from that of the Southern of Iran, 8 compounds (84.38%) from that of Pakistan, 48 compounds (75.3%) from that of India and 13 compounds (91%) from that of Bulgaria^7,8,10,15–17^.

**Table 1:** The chemical composition of essential oil of *R. damascena.*

No	Compound	RI*	RI	Mean (%) ± SD	Molecular formula
1	α-Pinene	881.280	882	0.47 ± 0.04^o^	C_10_H_16_
2	Linalool	1027.248	1026	0.89 ± 0.02^ l^	C_10_H_18_O
3	Citronellol	1120.192	1123	12.61 ± 0.01^d^	C_10_H_20_O
4	Geraniol	1135.576	1138	1.81 ± 0.01^ h^	C_10_H_18_O
5	Eugenol	1209.715	1210	1.12 ± 0.00^j^	C_10_H_12_O_2_
6	Methyleugenol	1229.383	1230	1.80 ± 0.02^ h^	C_11_H_14_O_2_
7	α-Guaiene	1240.521	1241	0.65 ± 0.01^n^	C_15_H_24_
8	α-Humulene	1253.791	1254	0.43 ± 0.00^0^	C_15_H_24_
9	Germacrene D	1269.905	1270	1.42 ± 0.00^i^	C_15_H_24_
10	δ-Guaiene	1281.753	1281	0.78 ± 0.02^ m^	C_15_H_24_
11	Hexadecane	1381.355	1327	0.80 ± 0.03^ m^	C_16_H_34_
12	1-Nonadecene	1478.811	1475	2.30 ± 0.01^ g^	C_19_H_38_
13	Nonadecane	1489.420	1489	24.72 ± 0.01^a^	C_19_H_40_
14	Eicosane	1538.684	1538	2.33 ± 0.03^ g^	C_20_H_42_
15	Palmitic acid (Hexadecanoic acid)	1545.263	1544	4.49 ± 0.03^e^	C_16_H_32_O_2_
16	Henicos-1-ene	1575.789	1584	1.03 ± 0.00^ k^	C_21_H_42_
17	Heneicosane	1592.367	1591	19.32 ± 0.00^b^	C_21_H_44_
18	Oleic Acid (9-Octadecenoic acid)	1638.781	2140	17.63 ± 0.00^c^	C_18_H_34_O_2_
19	Stearic acid (Octadecanoic acid)	1646.814	2172	4.09 ± 0.02f.	C_18_H_36_O_2_
20	1,19-Eicosadiene	1657.063	1600	1.13 ± 0.03^j^	C_20_H_38_
	Total			99.72	
	Monoterpenes hydrocarbons			0.47	
	Oxygenated monoterpenes			15.31	
	Sesquiterpenes hydrocarbons			3.28	
	Oxygenated sesquiterpenes			0	
	Others (Nonterpenoids)			80.66	

RI refers to the retention index identified by database NIST 014; RI*refers to the retention index calculated from the retention time relative to that of C8 – C40 n-alkanes; Values with different letters are statistically different (Duncan, *p* ≤ 0.05).

Nonterpenoids were those detected in the highest percentage (80.66%) in rose essential oil, while oxygenated sesquiterpenes were not present in it at all. In India, nonterpenoids with the amount of more than 60% and oxygenated sesquiterpenes in amount lower than 0.5% constituted the lowest and highest percentages respectively, of rose essential oils. Verma et al. showed that oil content, type, and oil composition varied among Damascus rose cultivars^16^. Content and composition of essential oil are complex traits that depend on yield components and have been strongly influenced by genetic and environmental factors. Thus, evaluating genotypes from different environments is an important step in Damascus's rosewood breeding programs before selecting the optimum for commercial cultivation. The yields and effects of medicinal plants vary depending on the locations of growth, climatic and ecological conditions, field operations, growth stages, and genetic traits^18–20^.

Nonadecane (24.72%) and heneicosane (19.32%) were the main constituents of the essential oil, in agreement with the study of Verma et al.^17^. Nonadecane (16.79–40.5%) was reported as the second dominant compound and heneicosane (7–14%) as the fourth dominant compound in central Iran^7,21^. The environmental and ecological factors in central Iran appear to be similar to those of India and are one of the influential factors in increasing the composition of aliphatic hydrocarbons, which reduces the aroma of rose essential oil.

Oleic acid with the amount of 17.63% was the third dominant component of rose essential oil, which was only reported previously in New Delhi with the amount of 0.37%^22^. This fatty acid has not been previously recorded in rose essential oils in Iran, while in this essential oil has been detected in high percentage, indicating a specific genotype of Iranian rose in the world. Oleic acid (9-Octadecenoic acid) is a component of omega-9 unsaturated fatty acids that is beneficial for the human body^23^. Indeed, it is well known that unsaturated fatty acids reduce cholesterol by activating cholesterol acetyltransferase^24^. Fatty acid has a great role in the treatment of cancer and cardiovascular, autoimmune, Parkinson's, alzheimer's, inflammatory and hypertension diseases. Its derivatives have a regulatory role on the cell membrane and have been used as an anticancer drug as they may induce apoptosis in cancer cells^25–27^. Also, two saturated fatty acids of stearic acid (4.09%) and palmitic acid (4.49%) were found in the essential oil that has not been previously detected in any of the essential oil from rose in the world. Palmitic and stearic acids are considered as precursors for the production of unsaturated fatty acids such as oleic acid. In other words, saturated fatty acids such as palmitic acid and stearic acid may be converted in oleic acid by enzymatic activity^28–30^.

Citronellol (12.61%) was one of the most abundant constituents of this essential oil, which plays an important role in its aroma. This compound has been detected in high amount also in the essential oil obtained from plants grown in central Iran (48.2%) and in Turkey (38.7%)^12,31^. Another important aromatic compound contained in the rose essential oil is geraniol (1.81%), which amount is much lower than that contained in the essential oil obtained in the central part of Iran (17%)^31^. But it should be noted that for a high quality essential oil, the ratio of citronellol/geraniol should be between 1.25 and 3.1%^32^, while it is 6.96% in the present study.

Eugenol was another important constituent of rose essential oil (1.12%) as well, which was detected in similar amount in the essential oil obtained in the central part of Iran (1%) and Pakistan (1.68%)^7,15^. This compound has an anticonvulsant effect^33^. Methyl-eugenol a natural carcinogenic compound with high antibacterial activity (1.80%) is also contained in rose essential oil^34^. The amount of linalool was around 0.8%, while a higher value was detected in the essential oil obtained from plants collected from the central part of Iran (1.97%) and the highest amount was found in the essential oil from the Saudi Arabia (8%)^7,35^. The antimicrobial and antiseptic properties of this compound have been confirmed in the study by Aali et al.^36^. According to the main constituents of the essential oil, it can be concluded that the studied sample is a specific genotype of rose in Isfahan province of Iran, which in addition to proper aroma, has unsaturated fatty acids with beneficial properties capable of promoting the human health.

### Antimicrobial effectiveness of essential oil

The antibacterial and antifungal properties of essential oil isolated from *R. damascena* flower was evaluated by measuring the inhibition halo diameter, the MIC, and the MBC. Results are presented in Table 2. The highest inhibition halo of rose essential oil was against *A. brasiliensis* (15.00 ± 0.00 mm), as its antifungal activity was higher that obtained by treating the same strain with nystatin (30 mm), while the inhibitory and lethal effects of the essential oil against this fungus were not significant. Interestingly, the antifungal activity of rose oil has not been previously studied in any area of the world, therefore, there was no study to compare the obtained results. This antifungal property can be connected with the activity of oxygenated compounds such as linalool^37^. The antimicrobial effect of the essential oil is not only due to their major constituents. Indeed, it is also possible that compounds in lower amount are likely to have synergistic effects with other active compounds^38^ promoting their effects. *Aspergillus* infections can occur in a variety of forms, including disease in hosts with normal immunity, infection in damaged host tissues, and infection in suppressed hosts^39^. On the other hand, due to the presence of degrading enzymes in these fungi, they can cause a high degree of corruption when they are on or inside the food, threatening human and animal health. Therefore, rose oil in the present study is a good natural source against the growth of *A. brasiliensis* and it prevents human diseases as well as food spoilage.

**Table 2:** Inhibition-zone diameters, Minimal inhibitory concentrations (MIC) and Minimal bactericidal/fungicidal (MBC) concentrations of essential oil of *R. damascena* and referent antibiotics.

Bacterial/fungal strains	Rose oils			Rifampin		Gentamicin		Nystatin	
	DD	MIC	MBC	DD	MIC	DD	MIC	DD	MIC
*S. aureus*	11.33 ± 0.58^b^	500 ± 0.00	> 1000 ± 0.00^e^	21 ± 0.00^a^	21.35 ± 0.00	21 ± 0.00^a^	1.95 ± 0.00^a^	NA	NA
*S. epidermidis*	ND	250 ± 0.00^c^	1000 ± 0.00^d^	27 ± 0.00^a^	1.95 ± 0.00^a^	27 ± 0.00^a^	1.95 ± 0.00^a^	NA	NA
*S. pyogenes*	9.33 ± 0.58^b^	250 ± 0.00^c^	250 ± 0.00^b^	21 ± 0.00^a^	0.975 ± 0.00^a^	21 ± 0.00^a^	0.975 ± 0.00^a^	NA	NA
*B. subtilis*	ND	500 ± 0.00^c^	> 1000 ± 0.00^e^	19 ± 0.00^a^	31.25 ± 0.00^b^	19 ± 0.00^a^	3.90 ± 0.00^a^	NA	NA
*Sh. dysenteriae*	ND	250 ± 0.00^c^	250 ± 0.00^b^	9 ± 0.00^a^	15.36 ± 0.00^b^	9 ± 0.00^a^	3.90 ± 0.00^a^	NA	NA
*P. aeruginosa*	ND	250 ± 0.00^c^	250 ± 0.00^b^	ND	31.25 ± 0.00^b^	ND	7.81 ± 0.00^a^	NA	NA
*E. coli*	ND	250 ± 0.00^c^	500 ± 0.00^c^	10 ± 0.00^a^	15.36 ± 0.00^a^	10 ± 0.00^a^	31.25 ± 0.00^b^	NA	NA
*K. pneumoniae*	8.00 ± 0.00^a^	500 ± 0.00^c^	500 ± 0.00^c^	8 ± 0.00^a^	15.36 ± 0.00^b^	8 ± 0.00^a^	3.90 ± 0.00^a^	NA	NA
*S. paratyphi-A*	ND	250 ± 0.00^c^	250 ± 0.00^b^	8 ± 0.00^a^	15.36 ± 0.00^b^	8 ± 0.00^a^	3.90 ± 0.00^a^	NA	NA
*C. albicans*	ND	125 ± 0.00^b^	125 ± 0.00^a^	NA	NA	NA	NA	33 ± 0.00^a^	125 ± 0.00^b^
*A. brasiliensis*	15.00 ± 0.00^c^	1000 ± 0.00^d^	1000 ± 0.00^d^	NA	NA	NA	NA	30 ± 0.00^a^	31.2 ± 0.00^b^

*DD* the diameters of the inhibition zones includes the diameters of disks (6 mm), *ND* not determined, *NA* no activity; Values with different letters are statistically different (Duncan, *p* ≤ 0.05).

The inhibition halo of the essential oil against *C. albicans* was below 6 mm (ND), and a significant inhibition and lethal effect (MIC and MBC = 125 μg/mL) were also observes, which was similar to the nystatin antibiotic (125 μg/mL). In comparison with the essential oil of rose in Bulgaria in the study of Gochev et al.^10^ and Jirovetz et al.^12^ with MIC value of 1024–600 μg/mL, the effect of Iranian essential oil is significant. The high content of nonadecane and heneicosane alkanes in rose oil seems to be one of the most important factors of its effective antifungal activity. Previous studies indicate a high percentage of alkanes such as nonadecane in essential oils with improve the antimicrobial activity^40^. Geraniol, on the other hand, is one of the contributing factors to its antifungal activity. The antifungal effect of geraniol on *C. albicans* has been previously demonstrated by Jirovetz et al.^12^. Although α-pinene is present in lower amount in rose essential oils, it may be one of the contributing factors for its antifungal activity as already reported by Dorman and Deans^41^. In general, the synergistic effects of the different components of the essential oil on their biological and antimicrobial activity should be considered^42–44^. Predisposing factors such as widespread and prolonged use of antibiotics, corticosteroids, immunosuppressive drugs and underlying diseases such as diabetes, leprosy, and malignancies have caused the increase of fungal diseases, especially candidiasis, in the past. In some studies, *Candida* species accounted for the fourth leading cause of death from circulatory infections and accounted for 35% of deaths from bloodstream infections^45–47^. *C. albicans* causes many clinical manifestations, such as skin infection, endocarditis, vaginitis, cerebral abscess vaginitis, meningitis, endocarditis, pyelonephritis, arthritis inhuman^48^. So rose oil can be a natural alternative capable of preventing and/or inhibiting fungal infection.

Rose essential oil had a significant effect on Gram-negative bacterium *K. pneumonia*e with inhibition halo of 8.00 ± 0.00 mm, which was equal to that obtain by using rifampin and gentamicin (8 mm). However, the inhibitory and lethal power of the essential oil against this bacterium (MIC and MBC = 500 μg/mL) was not significant. But compared to the study of Jirovetz et al.^12^ on Bulgarian rose oil (diameter of inhibition zone of 10 mm and MIC of 600 μg/mL), the effect of rose oil in the present study was better. High levels of citronellol as well as the presence of geraniol in the rose essential oil seemed to be responsible for the inhibition of the growth of this bacterium. Some researchers reported that rose essential oils containing citronellol and geraniol had strong antimicrobial activity against some bacteria including this^12,49^. On the other hand, the presence of oleic acid, stearic acid, and palmitic acid are other compounds contributing to the antibacterial effect of this essential oil. Fatty acids have antifungal and antibacterial activities against many microorganisms whose spectrum of activity varies by degree of saturation, carbon-chain length and orientation of the double bond^50^. Although the mechanism of antibacterial activity of fatty acids is not yet known, it is believed that their functional nature is related to the permeability and membrane disruption and fatal alterations in the cytoplasmic content of the bacterium, thereby rupture/alteration of the membrane-dependent conduction systems may occur^51^. Carbapenemase-producing *K. pneumoniae* infections will be associated with increased treatment costs and prolonged hospitalization, treatment failure, and mortality. The poor prognosis of infections caused by Gram-negative bacteria producing carbapenemase has been reported. In a USA report on circulatory infections caused by carbapenemase-producing bacteria in 2011, patients reported a mortality rate of 47 to 66 percent. A study has shown that the risk of dying is twice as high in patients with infections caused by these strains^52^.

Rose essential oil had a lower activity against Gram-positive *S. aureus* (11.33 ± 0.58 mm) compared to rifampin and gentamicin (21 mm). However, the inhibitory and lethal power of the essential oil against these bacteria (MIC = 500 μg/mL and MBC = 1000 μg/mL) was not significant, which was less than the antibacterial activity of rose oil against this bacterium in Bulgaria (diameter of inhibition zone = 20 mm and MIC = 60 μg/mL), and Saudi Arabia (MIC = 250 μg/mL)^12,13,53^. It appears that citronellol and geraniol, and even a small amount of α-pinene are contributing factors in this activity. The inhibitory effect of citronellol and geraniol on this bacterium has been reported^12^. The purified α-pinene antimicrobial activity of *S. aureus* has been demonstrated^54^. *S. aureus* is one of the most important pathogens transmitted through food that is widely distributed in the environment and human and animal communities, both as pathogens and as normal flora. The presence of *S. aureus* in the skin and respiratory organs of human and warm animals allows the transmission of this organism from human or animal to food materials and products^55^.

Rose essential oil against Gram-positive *S. pyogenes* also produced inhibition halo of 9.33 ± 0.58 mm, which was relatively low compared to that obtained by treating the same bacteria with rifampin and gentamicin (21 mm) and had low inhibitory and lethal power against this bacterium (MIC and MBC = 250 μg/mL). Compared with the results of the study of Shohayeb et al.^13^ obtained by using the rose essential oil from Saudi Arabia (MIC = 250 μg/mL and MBC = 500 μg/mL), the essential oil obtained in this work had a stronger lethal effect. *S. pyogenes* seems to be a component of beta-hemolytic *Streptococcus* A. Monoterpene compounds such as α-pinene cause this weak activity. Studies of the inhibition halo of α-pinene on these bacteria have been documented. This group of *Streptococcus* are located in the throat and skin and are responsible for a variety of purulent infections and non-purulent consequences^32,56^. The effect of rose essential oil on the two Gram-positive bacteria, although not significant, could be one of the side effects along with other naturally occurring high-impact compounds and could be used in the manufacture of natural antimicrobials.

## Conclusions

Rose essential oil is an extractive product with high medicinal and economic value due to its fragrance quality. In the present study, on the one hand, citronellol and geraniol compounds produced a high quality aroma of this essential oil, and on the other hand, probably the prevalence of nonadecane and heneicosane alkanes and the presence of linalool caused strong antifungal activity against *A. brasiliensis* and *C. albicans*. Also, for the first time, saturated and unsaturated fatty acids such as oleic acid, stearic acid and palmitic acid were found in large amount in this essential oil. These compounds might had a strong effect against Gram-negative bacterium *K. pneumonia*, which should be the subject of the future studies. Therefore, this essential oil can be a potential natural compound for the prevention and treatment of fungal diseases in humans and food spoilage.
